# The impact of social cognition deficits on real life functioning in 22q11.2 deletion syndrome: A comparative study with a large population of patients with schizophrenia

**DOI:** 10.1192/j.eurpsy.2021.1445

**Published:** 2021-08-13

**Authors:** T. Accinni, M. Frascarelli, A. Buzzanca, F. Ghezzi, L. Carlone, A. Panzera, A. Moschillo, N. Girardi, F. Di Fabio

**Affiliations:** Department Of Human Neurosciences Sapienza University Of Rome, Viale dell’Università 30, Rome, Italy

**Keywords:** 22q11.2 Deletion Syndrome, social cognition, schizophrénia, Real life functioning

## Abstract

**Introduction:**

22q11.2 Deletion Syndrome (22q11.2DS) represents a congenital syndrome with several clinical features. It entails a 25% risk of psychotic onset in lifespan. 22q11.2DS is a reliable model for biological vulnerability to schizophrenia.

**Objectives:**

With the hypothesis of similar impairments in schizophrenia and 22q11DS, to investigate a possible correlation between Social Cognition (SC) and Interpersonal Functioning (FU).

**Methods:**

Sample consists of 1735 adults: 893 schizophrenic subjects (SCZ); 18 with 22q11.2DS and psychosis (DEL_SCZ); 44 22q11.2DS individuals (DEL); 780 healthy controls (HC). SCZ and HC data come from a multicentric study by Network for Research on Psychoses. SC was assessed with The Awareness of Social Interference Test (TASIT, consisting of three sections: T1= Emotion Recognition; T2=Minimal Social Inference; T3=Social Inference Enriched). The Specific Levels of Functioning (SLOF) interview was employed.

**Results:**

DEL_SCZ (p<0.001) and SCZ (p<0.001) showed impairments in each TASIT sections compared to HC. Significant deficits in interpersonal functioning area were found in SCZ (p<0.001) compared to HC. The interpersonal functioning domain showed a positive correlation with SC in HC (T1: r=0.097; p<0.001; T2: r=0.120; p=0.001; T3: r=0.121; p=0.001); DEL (T1: r=0.380; p=0.024; T2: r=0.466; p=0.005) and SCZ (T1: r=0.113, p=0.001; T2: r=0.110, p=0.001; T3: r=0.134; p<0.001).

**Conclusions:**

SC deficits both in subjects with 22q11.2DS and in people with schizophrenia suggest a role of endophenotypes. SC is directly correlated to interpersonal functioning in 22q11.2DS without psychosis and people with schizophrenia. DEL_SCZ may suffer from deeper cognitive and symptomatic conditions that both impact differently on FU.

